# Homogenization of two fluid flow in porous media

**DOI:** 10.1098/rspa.2014.0564

**Published:** 2015-04-08

**Authors:** K. R. Daly, T. Roose

**Affiliations:** Faculty of Engineering and Environment, University of Southampton, Southampton SO17 1BJ, UK

**Keywords:** porous media, Richards' equation, homogenization

## Abstract

The macroscopic behaviour of air and water in porous media is often approximated using Richards' equation for the fluid saturation and pressure. This equation is parametrized by the hydraulic conductivity and water release curve. In this paper, we use homogenization to derive a general model for saturation and pressure in porous media based on an underlying periodic porous structure. Under an appropriate set of assumptions, i.e. constant gas pressure, this model is shown to reduce to the simpler form of Richards' equation. The starting point for this derivation is the Cahn–Hilliard phase field equation coupled with Stokes equations for fluid flow. This approach allows us, for the first time, to rigorously derive the water release curve and hydraulic conductivities through a series of cell problems. The method captures the hysteresis in the water release curve and ties the macroscopic properties of the porous media with the underlying geometrical and material properties.

## 1. Introduction

The macroscopic flow of multiple fluid phases in porous media, for example soil, is often described by Richards' equation [1–3]. This equation describes the local saturation under the influence of saturation and pressure gradients and is parametrized by the water release curve and the saturation-dependent hydraulic conductivity, both are measured experimentally [4]. Richards' equation offers challenges in terms of both parametrization [4–6] and the numerical solution [7,8].

Mathematically it has been shown, using the method of homogenization [9,10], that single phase flow in porous media can be approximated by Darcy's Law [11]. This equation can be derived from Stokes equations for single phase flow in the pore space and is parametrized by the hydraulic conductivity [3,11,12]. Such techniques have been applied in single porosity materials [3,11,13,14], dual porosity materials [15–17] and vuggy porous structures [18–22]. However, the homogenization process in partially saturated porous media is less well defined and relies on assumed knowledge of the interface location (ch. 5 in [3]). Knowledge of the air–water interface is often hard to obtain. Studies using X-ray computed tomography have been carried out [23]. However, these are computationally intensive and require scans to be carried out across the whole range of saturation. To overcome the need for repeated scanning, various researchers have suggested different empirical or approximate formula to describe the water release curve [2,3,24–26]. However, the water release curve exhibits multi-branched hysteresis loops [6] and needs to be parametrized with experimental measurements that can take months to gather even for a single branch of the hysteresis curve [5].

In order to derive the water release curve and saturation-dependent hydraulic conductivity, the dynamic interaction of the two fluid phases must be considered. One way to capture the physics of the two fluids is to use the Cahn–Hilliard phase field model coupled with Stokes equations [27–29]. The Cahn–Hilliard model can be derived through a free energy minimization [30,31] and has widely been applied to study moving contact lines and bubbles in a two fluid system [32]. This model overcomes the difficulties of a sharp interface by using the assumption that the interface between the two phases is of finite thickness which is assumed to be small in comparison with the geometry considered. By considering the limit when the interface thickness approaches zero the phase field model can be shown to reduce to standard free–boundary problems [32–34]. Homogenization of the Cahn–Hilliard model in porous media has previously only been studied for the case in which the interface thickness is comparable with the characteristic pore size [35,36]. This results in an effective Cahn–Hilliard equation where the interface mobility is derived through a set of cell problems.

In this paper, we consider the case of two immiscible fluids separated by an interface of finite width. This width is assumed small relative to the pore size and, hence, may be considered the smallest length scale in the problem. We use the method of homogenization to derive a coupled set of equations which describe the macroscopic flow properties of these fluids in partially saturated porous media. These equations, which are based on fundamental physical assumptions, are shown to reduce to Richards' equation in an appropriate parameter regime, i.e. the gas pressure is assumed constant. We assume that, to leading order, the interface positions and, hence, the water release curve are determined by capillary forces. The hydraulic conductivity is then determined by studying the perturbation due to a weak pressure gradient. The result is that the water release curve and the saturation-dependent hydraulic conductivity are determined through a series of cell problems which capture the porous geometry and the effects of the pressure gradients.

To capture the physics associated with two phase flow in porous media, the correct behaviour of the contact angle between the solid and the two fluid phases must be included in the Cahn–Hilliard formulation. The contact angle is one of the many factors associated with the hysteresis observed in the water release curve [37]. There have been numerous studies on the effect of the contact angle in the Cahn–Hilliard formulation [38–40]. Here, we make use of the more recent boundary conditions which are derived geometrically [40].

This paper is arranged as follows: in §2, we derive the Cahn–Hilliard fluid model using a free energy minimization and apply the method of homogenization to derive the two fluid model. The cell problems, which result from the calculations, allow the hydraulic conductivity and water release curves to be calculated based entirely on the underlying geometry. In §3, we study the solution properties of the cell problems and consider the limit when the interface thickness approaches zero. In §4, we solve the cell problems for the example case of air and water flowing in soil. Finally, in §5, we discuss our results and draw conclusions.

## 2. Derivation of homogenized equations

### (a) Deriving the two fluid Cahn–Hilliard equation

**Figure 1.**
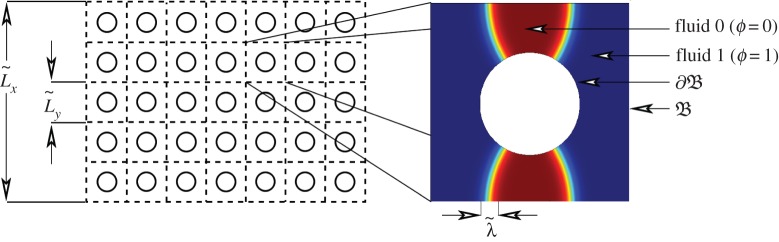
Schematic of porous domain (left) with length scales ${\tilde{L}}_{y}$, the scale of one periodic unit cell, and ${\tilde{L}}_{x}$, the macroscopic length scale. The box (right) shows a zoomed in view of one periodic unit cell of the fluid domain, *Ω*, the boundary of the porous structure ∂B and the two different fluids *ϕ*=0 and *ϕ*=1 with interface width $\tilde{\lambda }$. (Online version in colour.)

We consider the interaction of two single component fluids, for example air and water, in a porous geometry as illustrated in figure 1. Throughout this derivation we use $\tilde{\cdot }$ to denote that ⋅ is a dimensional quantity. A list of the dimensional quantities used in this derivation is given in table 1. We consider a porous domain *Ω* comprising a solid matrix S and a fluid part $B^{\epsilon }$ with boundary $\partial B^{\epsilon }$. We assume that $B^{\epsilon }$ is connected and that $\partial B^{\epsilon }$ is smooth. The geometry of the porous structure is of typical size ${\tilde{L}}_{x}$ and comprises a series of regularly repeating units of size ${\left(0,{\tilde{L}}_{y}\right)}^{3}$, where ${\tilde{L}}_{y}/{\tilde{L}}_{x}=\epsilon \ll 1$.

**Table 1.** Dimensional variables.

symbol	units	description
${\tilde{L}}_{x}$, ${\tilde{L}}_{y}$	m	macroscopic and microscopic length scales
$\tilde{\lambda }$	m	fluid–fluid interface thickness
${\tilde{\eta }}^{\left(0\right)}$, ${\tilde{\eta }}^{\left(1\right)}$, $\tilde{\eta }$	Pa s	viscosity of phase 0, phase 1 and the combined phase
${\tilde{\rho }}^{\left(0\right)}$, ${\tilde{\rho }}^{\left(1\right)}$	kg m^−3^	density of phase 0 and phase 1
$\tilde{\gamma }$	N m^−1^	surface tension at fluid–fluid interface.
${\tilde{F}}_{b}$, ${\tilde{F}}_{s}$, $\tilde{F}$	N m	bulk, surface and total free energies
${\tilde{\text{u}}}^{\left(0\right)}$, ${\tilde{\text{u}}}^{\left(1\right)}$, $\tilde{\text{u}}$	m s^−1^	velocity of phase 0, phase 1 and the combined phase
$\tilde{\zeta }$	kg m^−3^ s^−1^	fluid–fluid drag coefficient
$\tilde{p}$	kg m^−1^ s^−2^	combined fluid pressure
$\tilde{\sigma }$	kg m^−1^ s^−2^	combined stress tensor
$\tilde{R}$	N m s^−1^	Rayleighian
$\tilde{\mu }$	kg m^−1^ s^−2^	capillary pressure
$\tilde{t}$	s	time coordinate
$\tilde{\text{x}}$	m	space coordinate

To model the interaction between two fluids we use the Cahn–Hilliard model [27–29]. We define the phase field *ϕ*, a dimensionless variable that takes the value *ϕ*=1 in fluid 1 and *ϕ*=0 in fluid 0. At the interface between the two fluids *ϕ* changes smoothly from *ϕ*=0 to *ϕ*=1 over a distance $\tilde{\lambda }$ which we refer to as the interface thickness. We define the viscosity and density of fluid *j* as ${\tilde{\eta }}^{\left(j\right)}$ and ${\tilde{\rho }}^{\left(j\right)}$, respectively, for *j*={0,1}. We consider the case ${\tilde{\rho }}^{\left(1\right)}\gg {\tilde{\rho }}^{\left(0\right)}$ corresponding to, for example, air and water. However, we note that this is not a limitation of the model, it is instead used to simplify the notation and algebra used in the remainder of the paper.

To derive the equations that describe the interaction between these two fluids, we write an appropriate fluid free energy which we will then minimize [27–31]. Specifically we write the bulk and surface free energies of the fluid as

2.1 $${\tilde{F}}_{b}=\int_{B^{\epsilon }}\left[\alpha \tilde{\gamma }\left({\tilde{\lambda }}^{-1}f(\phi )+\frac{\tilde{\lambda }}{2}|\tilde{\nabla }\phi {|}^{2}\right)\right]d\tilde{x}\;\text{and}\;{\tilde{F}}_{s}=\int_{\partial B^{\epsilon }}\alpha \tilde{\gamma }h(\phi )\;d\tilde{x},$$

where *f*(*ϕ*)=*ϕ*^2^(1−*ϕ*)^2^, $\tilde{\gamma }$ is the surface tension and $\alpha =6\sqrt{2}$ scales the free energy such that $\tilde{\gamma }$ is the total excess free energy. It can be shown [40] that *h*(*ϕ*) satisfies $h^{\prime }(\phi )=\sqrt{2}\cos(\theta )\sqrt{f(\phi )}$, where *θ* is the contact angle between the fluid–fluid interface and the surface of the porous matrix which is assumed constant and *h*′(*ϕ*) is the variational derivative *δh*/*δϕ*. The fluid free energy consists of two terms: the bulk free energy which has minima at *ϕ*=0 and *ϕ*=1 and the interface energy which acts to minimize the total fluid volume over which *ϕ* is changing. We now proceed as in [30,31] and derive the momentum equations describing the fluid motion by minimizing the Rayleighian

2.2 $$\tilde{R}=\frac{\partial \tilde{F}}{\partial \tilde{t}}+\int_{B^{\epsilon }}\left[\frac{1}{2}\tilde{\zeta }{\left({\tilde{\text{u}}}^{\left(0\right)}-{\tilde{\text{u}}}^{\left(1\right)}\right)}^{2}+(\tilde{\sigma }-\tilde{p}-\tilde{\rho }{\tilde{\text{x}}}_{3}\tilde{g})\tilde{\nabla }\cdot \tilde{\text{u}}\right]d\tilde{x},$$

where $\tilde{F}={\tilde{F}}_{b}+{\tilde{F}}_{s}$ is the total fluid free energy, ${\tilde{\text{u}}}^{\left(0\right)}$ and ${\tilde{\text{u}}}^{\left(1\right)}$ are the fluid velocities of fluid 0 and 1, respectively, $\tilde{\text{u}}=\phi {\tilde{\text{u}}}^{\left(1\right)}+(1-\phi ){\tilde{\text{u}}}^{\left(0\right)}$ is the combined velocity, $\tilde{\zeta }$ is the drag coefficient between the two fluids, $\tilde{\sigma }=\tilde{\eta }[(\tilde{\nabla }\tilde{\text{u}})+{\left(\tilde{\nabla }\tilde{\text{u}}\right)}^{T}]$ is the combined stress tensor, $\tilde{g}$ is the acceleration due to gravity and $\tilde{\eta }(\phi )$ is the phase-dependent viscosity which takes the values $\tilde{\eta }(0)={\tilde{\eta }}^{\left(0\right)}$ and $\tilde{\eta }(1)={\tilde{\eta }}^{\left(1\right)}$ in fluid 0 and fluid 1, respectively. Similarly, $\tilde{\rho }(\phi )$ is the phase-dependent density which takes the values $\tilde{\rho }(0)={\tilde{\rho }}^{\left(0\right)}$ and $\tilde{\rho }(1)={\tilde{\rho }}^{\left(1\right)}$ in fluid 0 and fluid 1, respectively. Finally, $\tilde{p}$ is a Lagrange multiplier, effectively a combined or reduced fluid pressure, used to enforce incompressibility of the overall mixture. Assuming conservation of mass for each fluid, we also have

2.3 $$\frac{\partial \phi }{\partial \tilde{t}}=-\tilde{\nabla }\cdot (\phi {\tilde{\text{u}}}^{\left(1\right)})\;\text{and}\;\frac{\partial (1-\phi )}{\partial \tilde{t}}=-\tilde{\nabla }\cdot [(1-\phi ){\tilde{\text{u}}}^{\left(0\right)}].$$

Differentiating equation (2.1) with respect to time, using equation (2.3), and assuming the fluid velocity vanishes on the porous structure boundary, we find

2.4 $$\begin{array}{rl} \tilde{R} & =\int_{B^{\epsilon }}\frac{1}{2}\tilde{\zeta }{\left({\tilde{\text{u}}}^{\left(0\right)}-{\tilde{\text{u}}}^{\left(1\right)}\right)}^{2}+(\tilde{\sigma }-\tilde{p}-\tilde{\rho }{\tilde{\text{x}}}_{3}\tilde{g})\tilde{\nabla }\cdot \tilde{\text{u}}\;d\tilde{x}+\int_{B^{\epsilon }}\phi {\tilde{\text{u}}}^{\left(1\right)}\cdot \tilde{\nabla }\{\tilde{\gamma }\alpha [{\tilde{\lambda }}^{-1}f^{\prime }(\phi )-\tilde{\lambda }{\tilde{\nabla }}^{2}\phi ]\}\;d\tilde{x}\\ & \;+\int_{\partial B^{\epsilon }}[\alpha \tilde{\gamma }(\hat{\text{n}}\cdot \tilde{\lambda }\tilde{\nabla }\phi +h^{\prime }(\phi ))]\frac{\partial \phi }{\partial \tilde{t}}\;d\tilde{x}, \end{array}$$

where $\hat{\text{n}}$ is a unit vector normal to the surface of the porous medium. We minimize the Rayleighian with respect to $\tilde{p}$, ${\tilde{\text{u}}}^{\left(1\right)}$ and ${\tilde{\text{u}}}^{\left(0\right)}$ and, after some algebra and application of the divergence theorem, obtain the Cahn–Hilliard–Stokes system of equations

2.5a $$\begin{array}{rl} & \frac{\partial \phi }{\partial \tilde{t}}+\tilde{\nabla }\cdot (\phi \tilde{\text{u}})=\tilde{\nabla }\cdot \left\{\left[\frac{\phi^{2}{\left(1-\phi \right)}^{2}}{\tilde{\zeta }}\right]\tilde{\nabla }\tilde{\mu }\right\},\;\tilde{\text{x}}\in B^{\epsilon }, \end{array}$$

2.5b $$\begin{array}{rl} & \tilde{\nabla }\cdot \tilde{\sigma }-\phi \tilde{\nabla }\tilde{\mu }-\tilde{\nabla }\tilde{p}=\tilde{\rho }\tilde{g}{\hat{\text{e}}}_{3},\;\tilde{\text{x}}\in B^{\epsilon }, \end{array}$$

2.5c $$\begin{array}{r} \tilde{\zeta }({\tilde{\text{u}}}^{\left(0\right)}-{\tilde{\text{u}}}^{\left(1\right)})=(1-\phi )\phi \tilde{\nabla }\tilde{\mu },\;\tilde{\text{x}}\in B^{\epsilon }, \end{array}$$

2.5d $$\begin{array}{rl} & \tilde{\mu }=\alpha ({\tilde{\lambda }}^{-1}f^{\prime }(\phi )-\tilde{\lambda }{\tilde{\nabla }}^{2}\phi ),\;\tilde{\text{x}}\in B^{\epsilon }, \end{array}$$

2.5e $$\begin{array}{rl} \mathrm{and}\; & \tilde{\nabla }\cdot \tilde{\text{u}}=0,\;\tilde{\text{x}}\in B^{\epsilon }, \end{array}$$

combined with the no slip condition on the surface of the porous matrix

2.5f $$\tilde{\text{u}}=0,\;\tilde{\text{x}}\in \partial B^{\epsilon },$$

the contact angle boundary condition derived from (2.4)

2.5g $$\hat{\text{n}}\cdot \tilde{\lambda }\tilde{\nabla }\phi =-h^{\prime }(\phi ),\;\tilde{\text{x}}\in \partial B^{\epsilon }$$

and a zero flux condition to ensure the total volume of each phase is conserved

2.5h $$\hat{\text{n}}\cdot \left\{\left[\frac{\phi^{2}{\left(1-\phi \right)}^{2}}{\tilde{\zeta }}\right]\tilde{\nabla }\tilde{\mu }\right\}=0,\;\tilde{\text{x}}\in \partial B^{\epsilon }.$$

Here $\tilde{\mu }$ is the capillary pressure which is defined in equation (2.5d) and ${\hat{\text{e}}}_{3}$ is a unit vector in the direction ${\tilde{x}}_{3}$. We shall discuss the relationship between the capillary pressure and the specific fluid pressures in §2*c*. The additional condition, equation (2.5h), ensures the conservation of mass of each fluid on the boundary of the porous structure. Equations (2.5) combined with the initial condition $\phi (\tilde{\text{x}},0)=\bar{\phi }(\tilde{\text{x}})$ describe the two fluid behaviour in the porous structure.

### (b) Non-dimensional equations

We non-dimensionalize equations (2.5) by first scaling space with the microscopic length scale ${\tilde{L}}_{y}$ such that $\tilde{\text{x}}={\tilde{L}}_{y}\text{y}$ and $\nabla ={\tilde{L}}_{y}\tilde{\nabla }$. Using this scaling we define the unit cell *Y* =(0,1)^3^ composed of a fluid part B with boundary ∂B. We introduce the non-dimensional velocity $\text{u}={\left[\text{u}\right]}^{-1}\tilde{\text{u}}$, pressure $p={\left[p\right]}^{-1}\tilde{p}$, capillary pressure $\mu ={\left[\mu \right]}^{-1}\tilde{\mu }$ and time $t={\left[t\right]}^{-1}\tilde{t}$, where

2.6 $$[\text{u}]=\frac{\rho^{\left(1\right)}\tilde{g}{\tilde{L}}_{y}^{2}}{4{\tilde{\eta }}^{\left(1\right)}},\;[p]=\frac{[\text{u}]{\tilde{\eta }}^{\left(1\right)}}{{\tilde{L}}_{x}},\;[\mu ]=\frac{\alpha \tilde{\gamma }}{{\tilde{L}}_{y}},\;[t]=\frac{{\tilde{L}}_{y}}{\left[\text{u}\right]}.$$

We also introduce the dimensionless capillary number (*Ca*), Peclet number (*Pe*), Cahn number (λ) and scaled gravitational force (*g*):

2.7 $$Ca=\frac{{\tilde{L}}_{x}}{{\tilde{L}}_{y}}\frac{\eta^{\left(1\right)}[\text{u}]}{\alpha \tilde{\gamma }},\;Pe=\frac{{\tilde{L}}_{y}{\tilde{L}}_{x}\tilde{\zeta }[\text{u}]}{\alpha \tilde{\gamma }},\;\lambda =\frac{\tilde{\lambda }}{{\tilde{L}}_{y}},\;g=4\frac{{\tilde{\rho }}^{\left(1\right)}-{\tilde{\rho }}^{\left(0\right)}}{{\tilde{\rho }}^{\left(1\right)}},$$

where we have used ${\tilde{\rho }}^{\left(0\right)}/{\tilde{\rho }}^{\left(1\right)}∼O(\epsilon )$, physically this is equivalent to neglecting the influence of gravity on the phase *ϕ*=0. Finally, we define the phase-dependent viscosity

2.8 $$\eta (\phi )=\frac{{\tilde{\eta }}^{\left(0\right)}}{{\tilde{\eta }}^{\left(1\right)}}+\frac{{\tilde{\eta }}^{\left(1\right)}-{\tilde{\eta }}^{\left(0\right)}}{{\tilde{\eta }}^{\left(1\right)}}\phi .$$

We note that the definition of the Peclet number, which relates the diffusive motion of the interface to the advection by the fluid, is not, strictly speaking, a conventional Peclet number. However, as this is widely used in the literature we have chosen to keep this terminology [40,36]. Using these equations the scaled and dimensionless Cahn–Hilliard–Stokes equations are

2.9a $$\begin{array}{rl} & \frac{\partial \phi }{\partial t}+\text{u}\cdot \nabla \phi =\frac{1}{\epsilon Pe}\nabla \cdot M\nabla \mu ,\;\text{y}\in B, \end{array}$$

2.9b $$\begin{array}{rl} & \nabla \cdot \sigma -\frac{1}{\epsilon }\nabla p-\frac{1}{\epsilon \;Ca}\phi \nabla \mu =\phi g{\hat{\text{e}}}_{3}\;\text{y}\in B, \end{array}$$

2.9c $$\begin{array}{rl} & \nabla \cdot u=0\;\text{y}\in B, \end{array}$$

2.9d $$\begin{array}{rl} \mathrm{and}\; & \mu =\lambda^{-1}f^{\prime }(\phi )-\lambda \nabla^{2}\phi ,\;\text{y}\in B, \end{array}$$

where *σ*=*η*[(**∇*u***)+(**∇*u***)^*T*^] and *M*=*ϕ*^2^(1−*ϕ*)^2^. We solve these equations subject to the boundary conditions

2.9e $$\begin{array}{rl} & \text{u}=0,\;\text{y}\in \partial B, \end{array}$$

2.9f $$\begin{array}{rl} & \hat{\text{n}}\cdot \lambda \nabla \phi =-h^{\prime }(\phi ),\;\text{y}\in \partial B, \end{array}$$

2.9g $$\begin{array}{rl} \mathrm{and}\; & \hat{\text{n}}\cdot M\nabla \mu =0,\;\text{y}\in \partial B \end{array}$$

and the initial condition $\phi (\text{y},0)=\bar{\phi }(\text{y})$. Equations (2.9) form a complete non-dimensional description of the two fluid motion in the porous material. These will form the starting point of the homogenization procedure. We have scaled equations (2.9) such that *Ca*∼*O*(1) and *Pe*∼*O*(1) and the only small parameters in the final equations are *ϵ* and λ, i.e. the interface is narrow and we are considering a porous geometry with well-defined micro and macro scales. The result of the scaling given in equations (2.6) is that a unit change in *μ* drives a fluid velocity of order *ϵ*^−1^. This velocity corresponds to the movement of the fluid–fluid interface which decays rapidly to zero. Hence, the first non-zero contribution to the scaled velocity is order 1.

We are considering a problem with two different small parameters *ϵ*, the ratio of the microscopic and macroscopic length scales, and λ, the ratio of the interface thickness to the microscopic length scale, see figure 1. Before we proceed it is useful to discus the role of these two parameters. The first of these, *ϵ*, is standard in the homogenization literature [9] and will form the basis of the asymptotic expansion we will use in §2*c* to derive the averaged equations. The second small parameter λ is the non-dimensional interface thickness which must be small with respect to the minimum radius of curvature of the porous structure. We shall show in §3 that, in the limit λ→0, this model reduces to existing models where the fluid–fluid interface location is known (ch. 5 in [3]).

### (c) Homogenizing the Cahn–Hilliard fluid equation

We consider the geometry illustrated in figure 1. This geometry consists of a solid structure surrounded by pore space which contains two fluids. Our aim is to derive a set of macroscopic equations which are applicable on the length scale ${\tilde{L}}_{x}$ and describe the movement of these two fluids averaged over the length scale ${\tilde{L}}_{y}$, where ${\tilde{L}}_{y}/{\tilde{L}}_{x}=\epsilon \ll 1$. Due to the separation in length scales, and the periodicity of the geometry, the behaviour of the two fluids is, to first approximation, assumed periodic on the pore scale. Using this assumption and considering the solution to equations (2.9) in successive powers of *ϵ* we will derive a set of equations which describe the slow variation of these periodic solutions on the lengthscale ${\tilde{L}}_{x}$.

We define two different spatial coordinate systems; ***x*** denotes position on the macroscopic length scale and ***y*** denotes position on the microscopic length scale. The key assumption used in the following homogenization procedure is that these two length scales may be treated as independent [9]. Hence, we expand **∇**=**∇**_*y*_+*ϵ***∇**_*x*_, where **∇**_*x*_ denotes the gradient operator on the macroscopic length scale and **∇**_*y*_ denotes the gradient operator on the microscopic length scale. We also consider a set of different time scales *τ*_−1_=*ϵ*^−1^*t*, *τ*_0_=*t* and *τ*_1_=*ϵt*. These time scales correspond to the fast equilibration of the fluid–fluid interface, the medium time scale movement of the fluid–fluid interface on the scale ${\tilde{L}}_{y}$ and the slow variation in saturation due to applied pressure gradients, respectively.

Intuitively it may seem natural, as a first approximation, to neglect terms of order in λ and write λ in terms of *ϵ* before expanding as in, for example, [41]. However, if we do this, then the leading order solution is *ϕ*=const and multiple phases cannot co-exist. This is because the terms of order λ multiply the highest derivatives in equation (2.9) resulting in a singular perturbation scheme [42]. In order to accommodate the two phases there must be a region of thickness λ in which the function *ϕ* changes rapidly. As the interface position can change, it is not straightforward to construct an analytic solution in the interface region and match it to the solution in the regions of constant phase. Therefore, to leading order we must consider terms of order λ such that λ∇^2^*ϕ* balances λ^−1^*f*′(*ϕ*). Hence, we expand the velocity, pressure and phase only in powers of *ϵ*,

2.10 $$\text{u}={\text{u}}_{0}+\epsilon {\text{u}}_{1}+O(\epsilon^{2}),\;p=p_{0}+\epsilon p_{1}+O(\epsilon^{2}),\;\phi =\phi_{0}+\epsilon \phi_{1}+\epsilon^{2}\phi_{2}+O(\epsilon^{3}).$$

We also expand the stress tensor, *σ*=*σ*_0*y*_+*O*(*ϵ*), and the mobility *M*=*M*_0_+*ϵM*_1_+*O*(*ϵ*^2^), where

2.11a $$\begin{array}{rl} \sigma_{0y} & =(\nabla_{y}{\text{u}}_{0})+{\left(\nabla_{y}{\text{u}}_{0}\right)}^{T}, \end{array}$$

2.11b $$\begin{array}{rl} M_{0} & =M(\phi_{0})=\phi_{0}^{2}{\left(1-\phi_{0}\right)}^{2}, \end{array}$$

2.11c $$\begin{array}{rl} \mathrm{and}\;M_{1} & =\left({\frac{\delta M}{\delta \phi }|}_{\phi =\phi_{0}}\right)\phi_{1}=2\phi_{0}(1-\phi_{0})(1-2\phi_{0})\phi_{1} \end{array}$$

and *μ*=*μ*_0_+*ϵμ*_1_+*O*(*ϵ*^2^), where

2.11d $$\mu_{0}=\lambda^{-1}f^{\prime }(\phi_{0})-\lambda \nabla_{y}^{2}\phi_{0}$$

and

2.11e $$\mu_{1}=\lambda^{-1}f^{\prime \prime }(\phi_{0})\phi_{1}-\lambda (\nabla_{y}^{2}\phi_{1}+\nabla_{y}\cdot \nabla_{x}\phi_{0}+\nabla_{x}\cdot \nabla_{y}\phi_{0}).$$

We now substitute (2.10) and (2.11) into (2.9) and solve for ascending powers of *ϵ*.

#### (i) *O*(*ϵ*^−1^) problem

Substituting equations (2.11) into (2.9) and collecting terms of order *ϵ*^−1^ we obtain

2.12a $$\partial_{\tau_{-1}}\phi_{0}-Pe^{-1}\nabla_{y}\cdot M_{0}\nabla_{y}\mu_{0}=0,\;\text{y}\in B,$$

and

2.12b $$\nabla_{y}p_{0}+Ca^{-1}\phi_{0}\nabla_{y}\mu_{0}=0,\;\text{y}\in B,$$

with the boundary conditions

2.12c $$\hat{\text{n}}\cdot \lambda \nabla_{y}\phi_{0}=-h^{\prime }(\phi_{0}),\;\text{y}\in \partial B,$$

and

2.12d $$\hat{\text{n}}\cdot M_{0}\nabla_{y}\mu_{0}=0,\;\text{y}\in \partial B,$$

the initial condition $\phi_{0}(\text{x},\text{y},0)=\bar{\phi }(\text{y})$ and *p*_0_, *μ*_0_ and *ϕ*_0_ are periodic with period 1. Physically equations (2.12) describe the location of the fluid–fluid interface and are satisfied at steady state for any *μ*_0_ and *p*_0_ which are constant in ***y***, hence, $∥B∥\mu_{0}=\int_{B}\mu_{0}\;dy$, where $∥B∥=\int_{B}\;dy$. We note in passing that, at steady state, *ϕ*_0_ is a function of both ***x*** and ***y*** which we write as $\phi_{0}=S(\text{x})+\phi_{0}^{\left(m\right)}(\text{y})$ where $\phi_{0}^{\left(m\right)}$ is the modulated part of *ϕ*_0_ with zero average. Hence, the saturation is defined as

2.13 $$S=\frac{1}{∥B∥}\int_{B}\phi_{0}\;dy,$$

where *S* takes value *S*=1 for a fully saturated region and *S*=0 for a fully unsaturated region. Therefore, we write *μ*_0_∼*μ*_0_[*S*(***x***)], where *S*(***x***) varies only on the macroscopic scale.

Finally, we observe that, by defining the fluid pressure *p*_*s*_(***x***,***y***)=*p*_0_(***x***)+*Ca*^−1^*ϕ*_0_(***y***)*μ*_0_(***x***), we can rewrite equation (2.12b) as

2.14 $$\nabla_{y}p_{s}-Ca^{-1}\mu_{0}\nabla_{y}\phi_{0}=0.$$

Following the method outlined in [32] we integrate equation (2.14) over a cylinder of height 2*h*λ, where *h*≫1 centred about the interface and, after some algebra, obtain the Young–Laplace equation relating the capillary pressure to the pressure drop across the interface:

2.15 $${\hat{\text{n}}}_{\phi_{0}}p_{s}{|}_{\phi_{0}=1}-{\hat{\text{n}}}_{\phi_{0}}p_{s}{|}_{\phi_{0}=0}={\hat{\text{n}}}_{\phi_{0}}Ca^{-1}\mu_{0}.$$

Here ${\hat{\text{n}}}_{\phi_{0}}$ is a unit vector normal to the fluid–fluid interface. Hence we can write

2.16 $$p_{0}(\text{x})=p_{s}^{\left(0\right)}(\text{x})+\phi_{0}(p_{s}^{\left(1\right)}(\text{x})-p_{s}^{\left(0\right)}(\text{x})-Ca^{-1}\mu_{0}(\text{x})),$$

where $p_{s}^{\left(j\right)}$ is the specific pressure in the *j*th fluid and, using equation (2.15), we find $p_{0}=p_{s}^{\left(0\right)}$, i.e. *p*_0_ represents the specific pressure of phase 0.

In order to obtain a macroscopic theory which is valid for all saturation levels we will have to solve equations (2.12) for all possible initial saturation values. In reality this can be achieved using a discrete set of different saturation values and interpolating. It is also clear that the resulting value *μ*_0_(*S*) is dependent not only on the initial saturation, but also on the initial conditions, $\bar{\phi }$. For now we shall assume that we know $\bar{\phi }$ and, hence, *μ*_0_(*S*) can be determined and will revisit this point in §4.

#### (ii) *O*(*ϵ*^0^) problem

To proceed we collect terms of *O*(*ϵ*^0^) from the expansion of equation (2.9). First we consider the expansion of equation (2.9a) and the corresponding boundary condition (2.9g):

2.17a $$\begin{array}{rl} & \partial_{\tau_{-1}}\phi_{1}+\partial_{\tau_{0}}\phi_{0}+{\text{u}}_{0}\cdot \nabla_{y}\phi_{0}\\ & \;-Pe^{-1}[\nabla_{y}\cdot M_{0}\nabla_{y}\mu_{1}+\nabla_{y}\cdot M_{0}\nabla_{x}\mu_{0}+\nabla_{y}\cdot M_{1}\nabla_{y}\mu_{0}]=0,\;\text{y}\in B, \end{array}$$

and

2.17b $$\hat{\text{n}}\cdot M_{1}\nabla_{y}\mu_{0}+\hat{\text{n}}\cdot M_{0}\nabla_{y}\mu_{1}+\hat{\text{n}}\cdot M_{0}\nabla_{x}\mu_{0}=0,\;\text{y}\in \partial B.$$

Before we proceed we note that *μ*_0_∼*μ*_0_(***x***) such that **∇**_*y*_*μ*_0_=0 and the terms involving *M*_1_ in equations (2.17) vanish. We now check for solvability by integrating equations (2.17) over B and applying the divergence theorem. Hence, by the Fredholm alternative [9] for a solution to equations (2.17a) to exist, we require

2.18 $$\int_{B}(\partial_{\tau_{-1}}\phi_{1}+\partial_{\tau_{0}}\phi_{0})\;dy=0.$$

This is an equation for the *τ*_−1_ dependence of *ϕ*_1_ if we denote the volume average over the unit cell as $\langle \cdot \rangle =\int_{B}\cdot \;dy$ and integrate in *τ*_−1_ between 0 and *T*_−1_ we obtain

2.19 $$\langle \phi_{1}(T_{-1},\tau_{0},\ldots )\rangle -\langle \phi_{1}(0,\tau_{0},\ldots )\rangle =T_{-1}\partial_{\tau_{0}}\langle \phi_{0}(T_{-1},\tau_{0},\ldots )\rangle ,$$

where we have taken *T*_−1_≫1 such that *ϕ*_0_ has been in steady state for a long time. In order that *ϕ*_1_ does not grow linearly in time we require that ∂_*τ*_0__*ϕ*_0_=0 and, hence, *ϕ*_0_ is independent of *τ*_0_ and *ϕ*_1_ is independent of *τ*_−1_. The result is a set of equations for ***u***_0_, *p*_1_, *ϕ*_1_ and *μ*_1_:

2.20a $$\begin{array}{rl} & {\text{u}}_{0}\cdot \nabla_{y}\phi_{0}-Pe^{-1}[\nabla_{y}\cdot M_{0}\nabla_{y}\mu_{1}+\nabla_{y}\cdot M_{0}\nabla_{x}\mu_{0}]=0,\;\text{y}\in B, \end{array}$$

2.20b $$\begin{array}{rl} & \nabla_{y}\cdot \sigma_{0y}-\nabla_{y}p_{1}-\nabla_{x}p_{0}-Ca^{-1}[\phi_{0}\nabla_{y}\mu_{1}+\phi_{0}\nabla_{x}\mu_{0}]=\phi_{0}g{\hat{\text{e}}}_{3},\;\text{y}\in B, \end{array}$$

2.20c $$\begin{array}{rl} \mathrm{and}\; & \nabla_{y}\cdot {\text{u}}_{0}=0,\;\text{y}\in B, \end{array}$$

with boundary conditions

2.20d $${\text{u}}_{0}=0,\;\text{y}\in \partial B,$$

and

2.20e $$\hat{\text{n}}\cdot M_{0}\nabla_{y}\mu_{1}+\hat{\text{n}}\cdot M_{0}\nabla_{x}\mu_{0}=0,\;\text{y}\in \partial B,$$

the correction to the phase can be found using the following equation for *ϕ*_1_,

2.20f $$\mu_{1}=\lambda^{-1}f^{\prime \prime }(\phi_{0})\phi_{1}-\lambda (\nabla_{y}^{2}\phi_{1}+\nabla_{y}\cdot \nabla_{x}\phi_{0}+\nabla_{x}\cdot \nabla_{y}\phi_{0}),\;\text{y}\in B,$$

and

2.20g $$\hat{\text{n}}\cdot \lambda \nabla_{y}\phi_{1}+\hat{\text{n}}\cdot \lambda \nabla_{x}\phi_{0}+h^{\prime \prime }(\phi_{0})\phi_{1}=0,\;\text{y}\in \partial B,$$

and are ***u***_0_, *p*_1_, *μ*_1_ and *ϕ*_1_ periodic with period 1. We note, however, that we do not need to explicitly calculate *ϕ*_1_ in order to obtain the averaged equations. Equations (2.20) are linear in ***u***_0_, *p*_1_, *ϕ*_1_ and *μ*_1_ and depend on ***x***, ***y*** and *S*. Specifically *μ*_0_ is a function of saturation and, hence, ***x***, *p*_0_ is a function of ***x*** only and *ϕ*_0_ is a function of ***y*** and *S*. In order to find ***u***_0_, *p*_1_, *ϕ*_1_ and *μ*_1_ we consider the effects of the pressure and saturation gradients in equations (2.20) separately. We note that, as $\phi_{0}=S(\text{x})+\phi_{0}^{\left(m\right)}(\text{y})$ the terms of the form **∇**_*x*_⋅**∇**_*y*_*ϕ*_0_=0. We write the solution in the form

2.21 $$\begin{array}{rl} {\text{u}}_{0} & =\sum_{k=1}^{N}\kappa_{k}^{\mu }\partial_{x_{k}}\mu_{0}+\kappa_{k}^{p}\partial_{x_{k}}p_{0}+\kappa^{g}g,\;\mu_{1}=\sum_{k=1}^{N}\chi_{k}^{\mu }\partial_{x_{k}}\mu_{0}+\chi_{k}^{p}\partial_{x_{k}}p_{0}+\chi^{g}g,\\ \text{and}\;p_{1} & =\sum_{k=1}^{N}\omega_{k}^{\mu }\partial_{x_{k}}\mu_{0}+\omega_{k}^{p}\partial_{x_{k}}p_{0}+\omega^{g}g,\;\phi_{1}=\sum_{k=1}^{N}\psi_{k}^{\mu }\partial_{x_{k}}\mu_{0}+\psi_{k}^{p}\partial_{x_{k}}p_{0}+\psi^{g}g, \end{array}\}$$

where $\chi_{k}^{j}$, $\kappa_{k}^{j}$, $\omega_{k}^{j}$ and $\psi_{k}^{j}$ for *j*={*μ*,*p*,*g*} are assumed periodic with period 1. We note that the velocity written in the form of equation (2.21) is effectively a generalized Darcy's law. The functions $\chi_{k}^{\mu }$, $\kappa_{k}^{\mu }$, $\omega_{k}^{\mu }$ and $\psi_{k}^{\mu }$ satisfy the cell problem originating from **∇**_*x*_*μ*_0_

2.22a $$\begin{array}{rl} & \kappa_{k}^{\mu }\cdot \nabla_{y}\phi_{0}-Pe^{-1}[\nabla_{y}\cdot M_{0}\nabla_{y}\chi_{k}^{\mu }+\nabla_{y}\cdot M_{0}{\hat{\text{e}}}_{k}]=0,\;\text{y}\in B, \end{array}$$

2.22b $$\begin{array}{rl} & \nabla_{y}\cdot \sigma_{k}^{\mu }-\nabla_{y}\omega_{k}^{\mu }-Ca^{-1}\phi_{0}\nabla_{y}\chi_{k}^{\mu }=Ca^{-1}\phi_{0}{\hat{\text{e}}}_{k},\;\nabla_{y}\cdot \kappa_{k}^{\mu }=0,\;\text{y}\in B, \end{array}$$

2.22c $$\begin{array}{rl} & \kappa_{k}^{\mu }=0,\;\hat{\text{n}}\cdot M_{0}\nabla_{y}\chi_{k}^{\mu }+\hat{\text{n}}\cdot M_{0}{\hat{\text{e}}}_{k}=0,\;\text{y}\in \partial B, \end{array}$$

2.22d $$\begin{array}{rl} & \chi_{k}^{\mu }=\lambda^{-1}f^{\prime \prime }(\phi_{0})\psi_{k}^{\mu }-\lambda \nabla_{y}^{2}\psi_{k}^{\mu },\;\text{y}\in B, \end{array}$$

2.22e $$\begin{array}{rl} \text{and}\; & \hat{\text{n}}\cdot \lambda \nabla_{y}\psi_{k}^{\mu }=-h^{\prime \prime }(\phi_{0})\psi_{k}^{\mu },\;\text{y}\in \partial B, \end{array}$$

where $\sigma_{k}^{\mu }=\eta [(\nabla_{y}\kappa_{k}^{\mu })+{\left(\nabla_{y}\kappa_{k}^{\mu }\right)}^{T}]$. The functions $\chi_{k}^{p}$, $\kappa_{k}^{p}$, $\omega_{k}^{p}$ and $\psi_{k}^{p}$ satisfy the cell problem originating from **∇**_*x*_*p*_0_

2.23a $$\begin{array}{rl} & \kappa_{k}^{p}\cdot \nabla_{y}\phi_{0}-Pe^{-1}\nabla_{y}\cdot M_{0}\nabla_{y}\chi_{k}^{p}=0,\;\text{y}\in B, \end{array}$$

2.23b $$\begin{array}{rl} & \nabla_{y}\cdot \sigma_{k}^{p}-\nabla_{y}\omega_{k}^{p}-Ca^{-1}\phi_{0}\nabla_{y}\chi_{k}^{p}={\hat{\text{e}}}_{k},\;\nabla_{y}\cdot \kappa_{k}^{p}=0,\;\text{y}\in B, \end{array}$$

2.23c $$\begin{array}{rl} & \kappa_{k}^{p}=0,\;\hat{\text{n}}\cdot M_{0}\nabla_{y}\chi_{k}^{p}=0,\;\text{y}\in \partial B, \end{array}$$

2.23d $$\begin{array}{rl} & \chi_{k}^{p}=\lambda^{-1}f^{\prime \prime }(\phi_{0})\psi_{k}^{p}-\lambda \nabla_{y}^{2}\psi_{k}^{p},\;\text{y}\in B, \end{array}$$

2.23e $$\begin{array}{rl} \mathrm{and}\; & \hat{\text{n}}\cdot \lambda \nabla_{y}\psi_{k}^{p}=-h^{\prime \prime }(\phi_{0})\psi_{k}^{p},\;\text{y}\in \partial B, \end{array}$$

where $\sigma_{k}^{p}=\eta [(\nabla_{y}\kappa_{k}^{p})+{\left(\nabla_{y}\kappa_{k}^{p}\right)}^{T}]$. The remaining functions *χ*^*g*^, ***κ***^*g*^, *ω*^*g*^ and *ψ*^*g*^ satisfy the cell problem originating from *g*:

2.24a $$\begin{array}{rl} & \kappa^{g}\cdot \nabla_{y}\phi_{0}-Pe^{-1}\nabla_{y}\cdot M_{0}\nabla_{y}\chi^{g}=0,\;\text{y}\in B, \end{array}$$

2.24b $$\begin{array}{rl} & \nabla_{y}\cdot \sigma^{g}-\nabla_{y}\omega^{g}-Ca^{-1}\phi_{0}\nabla_{y}\chi^{g}=\phi_{0}{\hat{\text{e}}}_{3},\;\nabla_{y}\cdot \kappa^{g}=0,\;\text{y}\in B, \end{array}$$

2.24c $$\begin{array}{rl} & \kappa^{g}=0,\;\hat{\text{n}}\cdot M_{0}\nabla_{y}\chi^{g}=0,\;\text{y}\in \partial B, \end{array}$$

2.24d $$\begin{array}{rl} & \chi^{g}=\lambda^{-1}f^{\prime \prime }(\phi_{0})\psi^{g}-\lambda \nabla_{y}^{2}\psi^{g},\;\text{y}\in B, \end{array}$$

2.24e $$\begin{array}{rl} \mathrm{and}\; & \hat{\text{n}}\cdot \lambda \nabla_{y}\psi^{g}=-h^{\prime \prime }(\phi_{0})\psi^{g},\;\text{y}\in \partial B, \end{array}$$

where *σ*^*g*^=*η*[(**∇**_*y*_***κ***^*g*^)+(**∇**_*y*_***κ***^*g*^)^*T*^].

#### (iii) *O*(*ϵ*^1^) problem

We now expand the phase equation and the incompressibility condition to *O*(*ϵ*) and use the results of the previous expansions to write

2.25a $$\begin{array}{rl} & \partial_{\tau_{-1}}\phi_{2}+\partial_{\tau_{0}}\phi_{1}+\partial_{\tau_{1}}\phi_{0}+{\text{u}}_{1}\cdot \nabla_{y}\phi_{0}+{\text{u}}_{0}\cdot \nabla_{y}\phi_{1}+{\text{u}}_{0}\cdot \nabla_{x}\phi_{0}\\ & \;-Pe^{-1}[\nabla_{y}\cdot M_{0}(\nabla_{y}\mu_{2}+\nabla_{x}\mu_{1})+\nabla_{y}\cdot M_{1}(\nabla_{y}\mu_{1}+\nabla_{x}\mu_{0})\\ & \;+\nabla_{x}\cdot M_{0}(\nabla_{y}\mu_{1}+\nabla_{x}\mu_{0})+\nabla_{x}\cdot M_{1}\nabla_{y}\mu_{0}+\nabla_{y}\cdot M_{2}\nabla_{y}\mu_{0}]=0,\;\text{y}\in B, \end{array}$$

and

2.25b $$\nabla_{y}\cdot {\text{u}}_{1}+\nabla_{x}\cdot {\text{u}}_{0}=0,\;\text{y}\in B,$$

with the relevant boundary conditions

2.25c $${\text{u}}_{1}=0,\;\text{y}\in \partial B,$$

and

2.25d $$\hat{\text{n}}\cdot M_{0}(\nabla_{y}\mu_{2}+\nabla_{x}\mu_{1})+\hat{\text{n}}\cdot M_{1}(\nabla_{x}\mu_{0}+\nabla_{y}\mu_{1})+\hat{\text{n}}\cdot M_{2}\nabla_{y}\mu_{0}=0,\;\text{y}\in \partial B,$$

where *ϕ*_2_ and *μ*_2_ are periodic with period 1. As in the *O*(*ϵ*^0^) case the final terms in equation (2.25a) which contain multiples of **∇**_*y*_*μ*_0_ vanish as *μ*_0_∼*μ*_0_(***x***). Considering the solvability condition for equations (2.25), as in §2c(ii), we require that *ϕ*_1_ and *ϕ*_2_ do not grow linearly with time on any scale. Hence, the total fluid volume is conserved. Integrating equation (2.25a), applying the divergence theorem, and using equations (2.25b), (2.25c), (2.25d) with equations (2.20c) and (2.20d) we find, after some algebra,

2.26 $$∥B∥\frac{\partial S}{\partial \tau_{1}}-\nabla_{x}\cdot [a(S)K(S)\nabla_{x}S-b(S)\nabla_{x}p_{0}-b_{g}(S){\hat{\text{e}}}_{3}g]=0.$$

Similarly, we integrate equation (2.25b) over B and, using the divergence theorem, obtain

2.27 $$\nabla_{x}\cdot [a(S)\bar{K}(S)\nabla_{x}S-\bar{b}(S)\nabla_{x}p_{0}-{\bar{b}}_{g}(S){\hat{\text{e}}}_{3}g]=0,$$

where the saturation-dependent parameters in equation (2.26) are

2.28 $$\begin{array}{rl} a(S) & =-\frac{\delta \mu_{0}}{\delta S},\;K(S)=\int_{B}(\phi_{0}\kappa_{k}^{\mu }⊗{\hat{\text{e}}}_{k})\;dy+O(\lambda ),\\ \text{and}\;b(S) & =\int_{B}(\phi_{0}\kappa_{k}^{p}⊗{\hat{\text{e}}}_{k})\;dy+O(\lambda ),\;b_{g}(S)=\int_{B}(\phi_{0}\kappa^{g}⊗{\hat{\text{e}}}_{3})\;dy+O(\lambda ). \end{array}\}$$

We have simplified equations (2.28) by taking the limit λ→0. Hence, as *M*_0_ is only non-zero in a region of width λ, $\int_{B}M_{0}\;dy∼\lambda \to 0$. The parameters in equation (2.27) are

2.29 $$\bar{K}(S)=\int_{B}\kappa_{k}^{\mu }⊗{\hat{\text{e}}}_{k}\;dy,\;\bar{b}(S)=\int_{B}\kappa_{k}^{p}⊗{\hat{\text{e}}}_{k}\;dy,\;{\bar{b}}_{g}(S)=\int_{B}\kappa^{g}⊗{\hat{\text{e}}}_{3}\;dy.$$

Equations (2.26) and (2.27), with (2.28) and (2.29), form a pair of coupled equations for the macroscopic saturation and pressure. These are parametrized by the cell problems, equations (2.22), (2.23) and (2.24), which must be solved for a range of saturation.

We note that if *ϕ*_0_=1 everywhere, corresponding to full saturation, then $b(S)=\bar{b}(S)$ is the hydraulic conductivity of phase 1 and equations (2.26) and (2.27) become degenerate. Hence, we obtain Darcy's law for single phase flow. Similarly if *ϕ*_0_=0 everywhere then *b*(*S*)=0 and $\bar{b}(S)$ is the hydraulic conductivity of phase 0. Again, we obtain Darcy's law for single phase flow. By applying the assumptions used in deriving Richards' equation, i.e. that the pressure of phase 0 is constant in *Ω* we can write

2.30 $$∥B∥\frac{\partial S}{\partial \tau_{1}}-\nabla_{x}\cdot \{a(S)K(S)\nabla_{x}S+b_{g}(S){\hat{\text{e}}}_{3}g\}=0.$$

Equation (2.30) is the saturation form of Richards' equation which is valid assuming $\bar{K}(S)\ll \bar{b}(S)$ and ${\bar{b}}_{g}(S)\ll \bar{b}(S)$ such that equation (2.27) is approximately satisfied for constant pressure. We note that we could also have derived the mixed form of Richards' equation simply by leaving equations (2.26) and (2.27) in terms of *μ*_0_. The functions *ϕ*_0_, $\kappa_{k}^{\mu }$, $\kappa_{k}^{p}$ and ***κ***^*g*^*δμ*_0_/*δS* must be found for all saturation values. In reality it is enough to compute them for a subset of values and interpolate between them. The function *μ*_0_ is the scaled capillary pressure which is a function of geometry, contact angle and is history dependent. We investigate these effects using two and three dimensional examples in §4.

## 3. Analysis of homogenized equations

Equations (2.26) and (2.27) are valid across the whole range of saturation values. However, before we study the numerical solution of these equations we use matched asymptotics [42] to consider the limit of the Cahn–Hilliard–Stokes model when λ→0. It has been previously shown that, in this limit, the model reduces to standard free boundary problems for two fluid flow [32–34]. Here, in §3a, we use matched asymptotics to consider the limit of high and low saturation. Then, in §3b, we use these techniques to reduce the cell problems to those derived for fixed interfaces [3,22].

### (a) High and low saturation limit

Physically the high and low saturation limits correspond to the case where either the water or the air collects in the sampled geometry to form bubbles which are attached to the soil aggregate surface through capillary pressure. These bubble solutions are found by considering a single droplet of fluid attached to the porous structure. These droplets are assumed to be sufficiently small such that the surface to which they are attached may be considered planar, i.e. the radius of curvature of the droplet is much smaller than the radius of curvature of the porous structure. We start by following the method presented in [34] and considering a steady state radially symmetric solution to equations (2.11d), for *μ*_0_=*C* and C is a constant, in the absence of the porous structure. The solution is then patched onto the porous structure such that the contact angle which the droplet makes with the surface is correct and, hence, equations (2.12) are solved. Rewriting equation (2.11d) in radial coordinates we obtain

3.1 $$\lambda^{2}\left\{\frac{\partial^{2}\phi }{\partial r^{2}}+\frac{N-1}{r}\frac{\partial \phi }{\partial r}\right\}-f^{\prime }(\phi )=\lambda C,$$

where *N* is the number of dimensions considered, i.e. *N*=2 for 2D or *N*=3 for 3D. We consider the case of a bubble of dimensionless radius *r*_*b*_, where λ≪*r*_*b*_. We solve equation (3.1) using the method of matched asymptotics [42]. We define two outer regions, *r*<*r*_*b*_ and *r*>*r*_*b*_ and an inner region *r*∼*r*_*b*_. In order to obtain the full solution we are required to solve equation (3.1) in each region and match the solutions. However, as we are only interested in obtaining the value of the constant *C* in terms of radius, it is enough to consider only the leading order solution and the first order correction in the inner region.

We denote the solutions in the outer regions *ϕ*^(*o*−)^ for *r*<*r*_*b*_ and *ϕ*^(*o*+)^ for *r*>*r*_*b*_. The solution in the inner region is denoted *ϕ*^(*i*)^. Neglecting terms *O*(λ) we find *f*′(*ϕ*^(*o*−)^)=*f*′(*ϕ*^(*o*+)^)=0. For the inner region we rescale *ρ*=λ^−1^(*r*−*r*_*b*_) to obtain

3.2 $$\frac{\partial^{2}\phi^{\left(i\right)}}{\partial \rho^{2}}+\frac{\lambda (N-1)}{r_{b}-\lambda \rho }\frac{\partial \phi^{\left(i\right)}}{\partial \rho }-f^{\prime }(\phi^{\left(i\right)})=\lambda C,$$

with the boundary conditions *ϕ*^(*i*)^→*ϕ*^(*o*−)^ as ρ→−∞ and *ϕ*^(*i*)^→*ϕ*^(*o*+)^ as ρ→∞. We expand $\phi^{\left(i\right)}=\phi_{0}^{\left(i\right)}+\lambda \phi_{1}^{\left(i\right)}+O(\lambda^{2})$ and substitute into equation (3.2) to obtain the planar interface solution at leading order, i.e. $\phi_{0}^{\left(i\right)}$ satisfies

3.3 $$\frac{\partial^{2}\phi_{0}^{\left(i\right)}}{\partial \rho^{2}}-f^{\prime }(\phi_{0}^{\left(i\right)})=0,$$

with $\phi_{0}^{\left(i\right)}\to \phi^{\left(o-\right)}$ as ρ→−∞ and $\phi_{0}^{\left(i\right)}\to \phi^{\left(o+\right)}$ as ρ→∞. Solving for $\phi_{0}^{\left(i\right)}$ we find

3.4 $$\phi_{0}^{\left(i\right)}=\frac{1}{2}\left[1+\tanh\left(\frac{\rho }{\sqrt{2}}\right)\right].$$

Expanding to order λ we obtain

3.5 $$\frac{\partial^{2}\phi_{1}^{\left(i\right)}}{\partial \rho^{2}}-f^{\prime \prime }(\phi_{0}^{\left(i\right)})\phi_{1}^{\left(i\right)}=C-\frac{\left(N-1\right)}{r_{b}}\frac{\partial \phi_{0}^{\left(i\right)}}{\partial \rho },$$

with $\phi_{1}^{\left(i\right)}\to \phi_{1}^{\left(o\pm \right)}$ for ρ→±∞. Equation (3.5) is a linear equation of the form $L\phi_{1}^{\left(i\right)}=S$. In order that a solution exists we require that 〈*ψ*,*S*〉=0 for $\psi \in \text{ker}(L^{†})$, where the superscript † denotes the adjoint. In this case L is self adjoint and its kernel is spanned by the function $\partial \phi_{0}^{\left(i\right)}/\partial \rho$. Therefore, after some algebra, we find

3.6 $$\mu_{0}=\frac{\sqrt{2}(N-1)}{6r_{b}}+O(\lambda^{2}).$$

The partial bubble solution, where the fluid droplet is attached to a planar surface with contact angle *θ*, can be derived using simple geometric arguments. The droplet is assumed to be a partial sphere of volume V=S∥B∥ for low saturation and V=(1−S)∥B∥ for saturation close to 1 hence

3.7 $$r_{b}={\left[\frac{3V}{2+3\sin\theta -\sin^{3}\theta }\right]}^{1/3}.$$

We will make use of the bubble solution as the limit of high and low saturation in §4.

### (b) Comparison with existing models

We now compare our model with the existing two fluid homogenized models which are, for example, presented in ch. 5 in [3]. In these models the interface location is assumed to be known and the interface width is zero. The fluid velocity at the interface is assumed to be continuous in the directions tangential to the interface and zero in the direction normal to it. Finally, these equations are closed by assuming an unknown capillary pressure across the interface. These models can be homogenized using standard methods presented in, for example, [9]. The resulting cell problems can then be used to determine the macroscopic flow properties. In order to compare the equations we have derived to these models we consider the limit of (2.22), (2.23) and (2.24), as λ→0.

We consider cell problem (2.22) in detail as the same procedure can be applied easily to the cell problems (2.23) and (2.24). Far from the interface *M*_0_=0 and equations (2.22) simplify to the Stokes problem

3.8a $$\begin{array}{rl} & \nabla_{y}\cdot \sigma_{k}^{\mu }-\nabla_{y}\omega_{k}^{\mu }=0,\;\nabla_{y}\cdot \kappa_{k}^{\mu }=0,\;\text{y}\in B_{0}, \end{array}$$

3.8b $$\begin{array}{rl} & \nabla_{y}\cdot \sigma_{k}^{\mu }-\nabla_{y}\omega_{k}^{\mu }=Ca^{-1}{\hat{\text{e}}}_{k},\;\nabla_{y}\cdot \kappa_{k}^{\mu }=0,\;\text{y}\in B_{1}, \end{array}$$

3.8c $$\begin{array}{rl} \mathrm{and}\; & \kappa_{k}^{\mu }=0\;\text{y}\in \partial B, \end{array}$$

where $B_{1}$ is the region in which *ϕ*_0_=1 and $B_{0}$ is the region in which *ϕ*_0_=0. We note that at this point $\chi_{k}^{\mu }$ and $\psi_{k}^{\mu }$ are undefined. Equations (3.8) require a pair of boundary conditions at the interface between $B_{0}$ and $B_{1}$. To determine these conditions we rescale space with the interface thickness, **∇**_*y*_=λ^−1^**∇**_*z*_, to obtain

3.9a $$\begin{array}{rl} & \lambda^{-1}\kappa_{k}^{\mu }\cdot \nabla_{z}\phi_{0}-Pe^{-1}[\lambda^{-2}\nabla_{z}\cdot M_{0}\nabla_{z}\chi_{k}^{\mu }+\lambda^{-1}\nabla_{z}\cdot M_{0}{\hat{\text{e}}}_{k}]=0,\;\text{z}\in B, \end{array}$$

3.9b $$\begin{array}{rl} & \nabla_{y}\cdot \sigma_{k}^{\mu }-\lambda^{-1}\nabla_{z}\omega_{k}^{\mu }-\lambda^{-1}Ca^{-1}\phi_{0}\nabla_{z}\chi_{k}^{\mu }=Ca^{-1}\phi_{0}{\hat{\text{e}}}_{k},\;\lambda^{-1}\nabla_{z}\cdot \kappa_{k}^{\mu }=0,\;\text{z}\in B, \end{array}$$

3.9c $$\begin{array}{rl} & \kappa_{k}^{\mu }=0,\;\hat{\text{n}}\lambda^{-1}\cdot M_{0}\nabla_{z}\chi_{k}^{\mu }+\hat{\text{n}}\cdot M_{0}{\hat{\text{e}}}_{k}=0,\;\text{z}\in \partial B, \end{array}$$

3.9d $$\begin{array}{rl} & \chi_{k}^{\mu }=\lambda^{-1}(f^{\prime \prime }(\phi_{0})\psi_{k}^{\mu }-\nabla_{z}^{2}\psi_{k}^{\mu }),\;\text{z}\in B, \end{array}$$

3.9e $$\begin{array}{rl} \mathrm{and}\; & \hat{\text{n}}\cdot \nabla_{z}\psi_{k}^{\mu }=g^{\prime \prime }(\phi_{0})\psi_{k}^{\mu },\;\text{z}\in \partial B. \end{array}$$

We find a balance by scaling $\omega_{k}^{\mu }=\lambda {\bar{\omega }}_{k}^{\mu }$ and $\chi_{k}^{\mu }={\bar{\chi }}_{k}^{\mu }\lambda$. In order to obtain the appropriate interface conditions, we integrate equation (3.9b) over a cylinder of volume *V* and height 2*h*λ, where *h*≫1, centred about the interface. Applying the divergence theorem and taking the limit λ→0 we obtain the condition

3.10 $${\hat{\text{n}}}_{\phi_{0}}\cdot [\sigma_{k}^{\mu }-I\omega_{k}^{\mu }]{|}_{\phi_{0}=1}-{\hat{\text{n}}}_{\phi_{0}}\cdot [\sigma_{k}^{\mu }-I\omega_{k}^{\mu }]{|}_{\phi_{0}=0}={\hat{\text{n}}}_{\phi_{0}}{\bar{\chi }}_{k}^{\mu },$$

where we recall ${\hat{\text{n}}}_{\phi_{0}}$ is a unit vector normal to the fluid–fluid interface and *I* is the identity matrix. Similarly, we integrate equation (3.9a) over *V* to obtain

3.11 $$\int_{V}\kappa_{k}^{\mu }\cdot \nabla_{z}\phi_{0}-Pe^{-1}[\nabla_{z}\cdot M_{0}\nabla_{z}{\bar{\chi }}_{k}^{\mu }+\nabla_{z}\cdot M_{0}{\hat{\text{e}}}_{k}]\;dz=0.$$

We denote the end facets of the cylinder *S*0 in the region *ϕ*_0_=0 and *S*1 in the region *ϕ*_0_=1 and the cylinder side surface Δ*S*. Applying the divergence theorem, using equation (3.9b) and *M*_0_(1)=*M*_0_(0)=0 gives us the condition

3.12 $$\int_{S1}\hat{\text{n}}\cdot \kappa_{k}^{\mu }\;dz-Pe^{-1}\int_{\Delta S}\hat{\text{n}}\cdot (M_{0}\nabla_{z}{\bar{\chi }}_{k}^{\mu }+M_{0}{\hat{\text{e}}}_{k})\;dz=0,$$

where we have used continuity of velocity to simplify the integrals. Finally, observing that variation in $\chi_{k}^{\mu }$ tangential to the boundary is small and may be neglected at this order we obtain the boundary condition

3.13 $${\hat{\text{n}}}_{\phi_{0}}\cdot \kappa_{k}^{\mu }{|}_{\phi_{0}=1}=0.$$

Hence, if we define the fluid–fluid interface as $\partial B_{I}$ we obtain the cell problem in the limit λ→0

3.14a $$\begin{array}{rl} & \nabla_{y}\cdot \sigma_{k}^{\mu }-\nabla_{y}{\bar{\omega }}_{k}^{\mu }=0,\;\nabla_{y}\cdot \kappa_{k}^{\mu }=0,\;\text{y}\in B_{0}, \end{array}$$

3.14b $$\begin{array}{rl} & \nabla_{y}\cdot \sigma_{k}^{\mu }-\nabla_{y}{\bar{\omega }}_{k}^{\mu }=Ca^{-1}{\hat{\text{e}}}_{k},\;\nabla_{y}\cdot \kappa_{k}^{\mu }=0,\;\text{y}\in B_{1} \end{array}$$

3.14c $$\begin{array}{rl} & \kappa_{k}^{\mu }=0,\;\text{y}\in \partial B, \end{array}$$

3.14d $$\begin{array}{rl} & {\hat{\text{n}}}_{\phi_{0}}\cdot [\sigma_{k}^{\mu }-I{\bar{\omega }}_{k}^{\mu }]{|}_{\phi_{0}=1}-{\hat{\text{n}}}_{\phi_{0}}\cdot [\sigma_{k}^{\mu }-I{\bar{\omega }}_{k}^{\mu }]{|}_{\phi_{0}=0}={\hat{\text{n}}}_{\phi_{0}}{\bar{\chi }}_{k}^{\mu },\;\text{y}\in \partial B_{I}, \end{array}$$

3.14e $$\begin{array}{rl} & {\hat{\tau }}_{q}\cdot \kappa_{k}^{\mu }{|}_{\phi_{0}=0}={\hat{\tau }}_{q}\cdot \kappa_{k}^{\mu }{|}_{\phi_{0}=1},\;\text{y}\in \partial B_{I} \end{array}$$

3.14f $$\begin{array}{rl} \mathrm{and}\; & {\hat{\text{n}}}_{\phi_{0}}\cdot \kappa_{k}^{\mu }{|}_{\phi_{0}=1}=0,\;\text{y}\in \partial B_{I}, \end{array}$$

where ${\hat{\tau }}_{q}$ for *q*={1,2} is a unit vector tangent to the interface $\partial B_{I}$. Similarly, we find that $\kappa^{g}=Ca\kappa_{3}^{\mu }$, $\omega^{g}=Ca\omega_{3}^{\mu }$ and $\chi^{g}=Ca\chi_{3}^{\mu }$ and we obtain the cell problem for the pressure driven velocity field

3.15a $$\begin{array}{rl} & \nabla_{y}\cdot \sigma_{k}^{p}-\nabla_{y}{\bar{\omega }}_{k}^{p}={\hat{\text{e}}}_{k},\;\nabla_{y}\cdot \kappa_{k}^{p}=0,\;\text{y}\in B_{0}, \end{array}$$

3.15b $$\begin{array}{rl} & \nabla_{y}\cdot \sigma_{k}^{p}-\nabla_{y}{\bar{\omega }}_{k}^{p}={\hat{\text{e}}}_{k},\;\nabla_{y}\cdot \kappa_{k}^{p}=0,\;\text{y}\in B_{1}, \end{array}$$

3.15c $$\begin{array}{rl} & \kappa_{k}^{p}=0,\;\text{y}\in \partial B, \end{array}$$

3.15d $$\begin{array}{rl} & {\hat{\text{n}}}_{\phi_{0}}\cdot [\sigma_{k}^{p}-I{\bar{\omega }}_{k}^{p}]{|}_{\phi_{0}=1}-{\hat{\text{n}}}_{\phi_{0}}\cdot [\sigma_{k}^{p}-I{\bar{\omega }}_{k}^{p}]{|}_{\phi_{0}=0}={\hat{\text{n}}}_{\phi_{0}}{\bar{\chi }}_{k}^{p},\;\text{y}\in \partial B_{I}, \end{array}$$

3.15e $$\begin{array}{rl} \text{and}\; & {\hat{\tau }}_{q}\cdot \kappa_{k}^{p}{|}_{\phi_{0}=0}={\hat{\tau }}_{q}\cdot \kappa_{k}^{p}{|}_{\phi_{0}=1},\;{\hat{\text{n}}}_{\phi_{0}}\cdot \kappa_{k}^{p}{|}_{\phi_{0}=1}=0,\;\text{y}\in \partial B_{I}. \end{array}$$

Equations (3.14) and (3.15) correspond to the cell problems derived for a fixed interface in [22]. They can also be derived directly from the microscale equations for a fixed interface presented, for example, in ch. 5 in [3]. Hence, we see that taking the limits with respect to λ and *ϵ* commute for a fixed interface location.

## 4. Example

In this section we solve the cell problems (2.12), (2.22) and (2.23) to obtain the macroscopic parameters used in equations (2.26) and (2.27). As shown in §3, in the limit λ→0, the solution to the cell problem (2.24) is proportional to the solution to (2.22), hence we do not consider the solution to this cell problem explicitly. We consider, as an example, the flow of air and water in soil.

We recall *μ*_0_ is the leading order capillary pressure which we derive for a simplified two-dimensional example. The advantage to studying a simplified two-dimensional geometry is that it allows us to easily relate the calculated properties of the capillary pressure to the geometrical features. However, in this case we are interested only in the capillary pressure as the air and water phases can each easily form a plug for a large range of saturation resulting in zero hydraulic conductivity. In order to understand how contact angle affects the capillary pressure, we do this for a range of contact angles. The air–water contact angle with soil has been measured to be approximately 90° [43]. Hence, we consider contact angles of 70°, 90° and 110° as this range of angles provides a clear picture of how the capillary pressure varies near 90°. We then consider a simplified three-dimensional geometry with 90° contact angle and obtain the capillary pressure and hydraulic conductivities.

We note that the solution to the equations (2.12) is dependent on the initial condition. Therefore, the interface location and the capillary pressure are history dependent. In order to accommodate this, we calculate the capillary pressure curves for increasing saturation. We start from the water bubble solution and integrate equations (2.12) to steady state. The saturation is then increased by weakly perturbing the solution and equations (2.12) are solved again using the perturbed solution as the initial condition. By slowly increasing the saturation till the air bubble solution is reached the whole capillary pressure curve can be calculated. The equations are implemented in comsol multiphysics 4.3 using a combination of coefficient form PDEs and computational fluid dynamics modules. The equations are solved using a direct PARDISO method on a single 16 core node of the Iridis 4 supercomputing cluster at the University of Southampton. For both the two-dimensional and the three-dimensional case, the total simulation time is less than 20 h.

### (a) Two-dimensional soil

**Figure 2.**
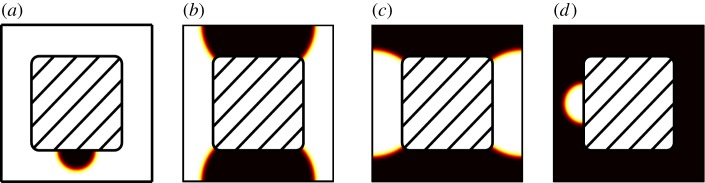
Interface position for increasing saturation: dark region is fluid 0 and light region is fluid 1. The geometry shown is periodic in the *x* and *y* directions with a shaded region representing a soil particle in the centre of the cell: (*a*) 95% saturation, (*b*) 55% saturation (*c*) 40% saturation and (*d*) 5% saturation. (Online version in colour.)

We derive water release curves, which show capillary pressure as a function of saturation, for the geometry shown in figure 2*a* for a range of different contact angles using the following method. We start by considering an initial partial air bubble attached to the soil. The size of the initial bubble is chosen to give 5% saturation. Equations (2.12) are solved to find the steady-state interface profile and, hence, value of *μ*_0_ corresponding to 5% saturation. The saturation is then increased by 1% and the process is repeated until 95% saturation is reached. The drying curve is captured by reversing the process and decreasing the saturation to 5%.

**Figure 3.**
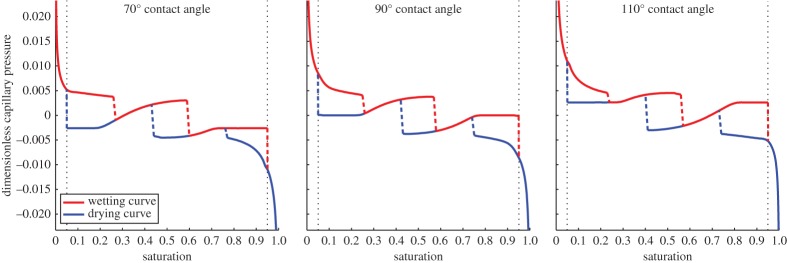
Water release curve for 70°, 90° and 110° contact angle, showing the wetting and drying curves for the three different contact angles. Each subplot shows three regions which are separated by the black dotted lines: R1 (the left most region) and R3 (the right most region) in which the bubble solution is valid and R2 (the middle region) which exhibits hysteresis and is strongly geometry dependent. (Online version in colour.)

The water release curve which results from this process is shown in figure 3 for contact angles of 70°, 90° and 110°. These curves are used to parametrize equations (2.26) and (2.27) through the parameter (2.28) and are valid on a timescale much slower than *τ*_−1_. The bubble solution, equation (3.6), is used for <5% or >95% saturation. The corresponding interface profiles as calculated using equations (2.12) are shown for the 90° contact angle in figure 2. It can be seen that at high and low saturation the water release curve follows the 1/*r*_*b*_ dependence that we would expect for a partial bubble solution.

In the geometry-dependent part of the water release curve, there are several discontinuities, shown with a dashed line. These jumps correspond to the saturation values at which the interface changes topology. As an example we follow the drying curve for the 90° contact angle in figure 3. At high saturation *S*>0.75, the interface forms a half bubble on the surface of the porous structure, see figure 2*a*. As the saturation is decreased below *S*=0.75 the volume of water contained in the bubble becomes to large to fit in the pore and the topology of the solution changes to the one shown in figure 2*b*. This solution remains valid for 0.75>*S*>0.43. For *S*<0.43 the solution topology changes again giving rise to the one shown in figure 2*c*. This solution remains valid until the air film becomes too thin and the solution collapses to the air bubble solution shown in figure 2*d*. In our simulations, we have taken this point to be *S*=0.95.

Increasing the contact angle away from normal incidence acts to increase the capillary pressure whilst maintaining the same set of topological solutions. The overall shape of the water release curve is unaffected by these changes.

### (b) Three-dimensional soil

For the three-dimensional case, the same algorithm is used as in the two-dimensional case to determine the water release curve. Once the steady-state interface location has been derived, the cell problems given in equations (2.22) and (2.23) are solved to obtain the hydraulic conductivities. We use ${\tilde{\eta }}^{\left(0\right)}=20\times 10^{-6}\;\text{Pa}\;\text{s}$ and ${\tilde{\eta }}^{\left(1\right)}=10^{-3}\;\text{Pa}\;\text{s}$ corresponding to the viscosity of air and water, respectively. For simplicity, we take *Ca*=*Pe*=1 and *θ*=90°.

**Figure 4.**
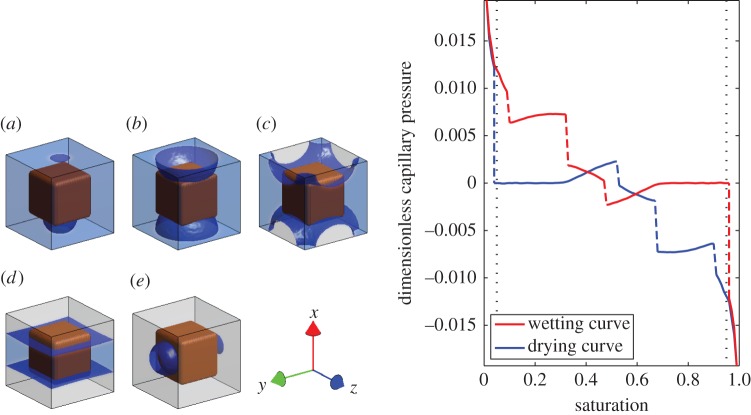
Interface position for decreasing saturation and the corresponding water release curve. The geometry shown is periodic in the *x*, *y* and *z* directions with a soil particle in the centre of the cell: (*a*) 95% saturation, (*b*) 75% saturation, (*c*) 55% saturation, (*d*) 35 % saturation and (*e*) 5 % saturation. The black dotted lines on the water release curve (right-hand side image) show 5% and 95% saturation, respectively. These correspond to the point at which the bubble solution is valid; this point is discussed further in the main text. (Online version in colour.)

**Figure 5.**
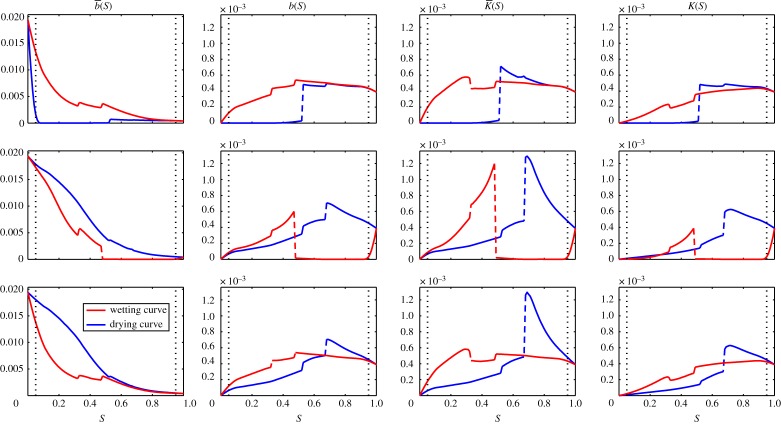
Effective diffusivity as a function of saturation for wetting and drying curves. Note the function $\bar{b}(S)$ uses a different scale to *b*(*S*), *K*(*S*) and $\bar{K}(S)$. The functions are calculated by solving equations (2.22) and (2.23) for 0.05<*S*<0.95 in steps of 0.01 and for *S*=0 and *S*=1. The values for 0<*S*<0.95 and 0.95<*S*<1 are calculated through interpolation. The black dotted lines show *S*=0.05 and *S*=0.95. (Online version in colour.)

The water release curve is calculated as in the two-dimensional case. Starting from the bubble solution at 95% saturation we find five topologically different solutions; these are shown in figure 4. The corresponding water release curve is also shown in figure 4 and the diffusivity curves, *K*(*S*), *b*(*S*), $\bar{K}(S)$ and $\bar{b}(S)$, are shown in figure 5.

As in the two-dimensional case, we see discontinuities in the water release curve where the interface location jumps between topologically different solutions. Following the drying curve we see that the bubble solution, figure 4*a*, is valid for a small range of saturation values *S*>0.91 before the droplet spans the gap between adjacent soil particles. At this point the solution topology changes to the one shown in figure 4*b*, which is valid for 0.91>*S*>0.68. In contrast to the two-dimensional case, an additional topological state is observed for 0.68>*S*>0.53, shown in figure 4*c*. This is observed when the air trapped between a pair of particles expands so much that it interacts with the air trapped between an adjacent pair of particles. As the saturation continues to decrease, the solution changes again to the one shown in figure 4*d* for 0.53>*S*>0.05 before collapsing to the bubble solution at low saturation *S*<0.05.

The corresponding diffusivity curves, neglecting terms of order λ, are plotted in figure 5 for the wetting and drying cycle. The functions *b*(*S*), *K*(*S*) and $\bar{K}(S)$ are zero for *S*=0 and increase to give the saturated hydraulic conductivity of the *ϕ*=1 phase at *S*=1. The function $\bar{b}(S)$ moves from the hydraulic conductivity of the *ϕ*=0 phase at *S*=0 to the hydraulic conductivity of the *ϕ*=1 phase at *S*=1.

As with the water release curve, the diffusivities are discontinuous corresponding to the solution switching between topologically different solutions. We note that the diffusivities are non-zero, providing that there is a connected flow pathway for either phase. To illustrate this, we follow the drying curves for all four diffusivities. At full saturation *S*=1 we obtain simply the diffusivity of phase 1 in all directions and all four diffusivity values are identical. Decreasing the saturation, we observe the half bubble solution figure 4*a* and the diffusivity in the *x* direction begins to differ from the solution in the *y* and *z* directions. Decreasing further we move through the solution shown in figure 4*b*,*c* with discontinuities visible in the diffusivity curves. These are most visible in the *K*(*S*) and $\bar{K}(S)$ curves; however, they are present in all four curves. In all four cases, the diffusivity increases as the lower viscosity phase, phase 0, acts to lubricate the flow of the higher viscosity phase, phase 1. At *S*=0.53 the solution changes to the one shown in figure 4*c*. At this point, neither phase 0 nor phase 1 is connected in the *x* direction and the diffusivity values all drop to zero in this direction. The corresponding values of *b*(*S*), $\bar{K}(S)$ and *K*(*S*) decrease monotonically from this point to 0 at *S*=0. The function $\bar{b}(S)$ increases monotonically from this point to reach the phase 0 diffusivity at *S*=0.

The value of $\bar{b}(S)$ is much larger than the other diffusivity curves and in the region 0.05<*S*<0.95 will dominate the behaviour of the flow. In the high and low saturation regimes, this will not be the case as *a*(*S*) grows rapidly will act to amplify the role of *K*(*S*) and $\bar{K}(S)$. Hence, in the intermediate saturation region, the final equations (2.26) and (2.27) could be simplified to neglect $\bar{K}(S)$ and ${\bar{b}}_{g}(S)$ such that equation (2.30) becomes valid under the constant pressure assumption.

## 5. Discussion

In this paper we have used the method of homogenization to derive, for the first time, a set of macroscopic equations for coupled saturation and pressure-driven fluid flow based entirely on the underlying geometry. The presence of multiple phases is described by the Cahn–Hilliard equation and fluid flow is governed by Stokes equations. The final equations are parametrized by the water release curve and four different diffusivity curves. These curves are captured by solving a series of different cell problems over a range of different saturation values. We have shown that these cell problems, and the resulting macroscopic equations, reduce to standard, simplified homogenizaiton models where the fluid–fluid interface is assumed to be known.

We have used several key assumptions in developing our model. The first is that the porous medium may be considered as a periodic structure. This approximation may be valid for man-made porous structures. However, for natural ones, such as soil, this is clearly only an approximation with the structure being quasi-periodic at best. This assumption has been tested for the case of two fluid flow in imaged soil geometries [23], for the case in which the air–water interface is obtained directly via imaging. In this case, the cell problems were solved on geometries of increasing size and the hydraulic properties were seen to converge. It is expected that the same will apply in this case and, hence, quasi-periodicity is enough for the model to remain valid. Secondly, based on the scaling used in equations (2.9) we require that the capillary forces dominate flow such that there is a separation in time scales between the movement of the fluid–fluid interface and the fluid velocity. This assumption is valid for sufficiently small pores as the fluid velocity shrinks with pore radius whilst the capillary pressure grows. Thirdly, we have modelled the interface between the two fluids using the Cahn–Hilliard equation in which the interface width is assumed non-zero. We have shown that, as the interface width tends to zero, the cell problems derived in §2a converge to those traditionally used for two fluid flow (ch. 5 in [3], [22]). For this approximation to be valid we have implicitly assumed that the interface width used in the numerical calculations is significantly less than the smallest geometrical feature observed. This assumption neglects the effects of small-scale surface roughness which could induce contact angle hysteresis. These effects could, in principle, be included through an effective contact angle-dependent on the small-scale surface geometry.

Using these assumptions we have been able to capture the main features of two fluid flow and, for a given periodic geometry, predict the water release and diffusivity curves. There are three distinct regions observed in these curves, the low- and high-concentration regimes, in which we find an approximate analytic solution for the water release curve, and an intermediate region, in which the water release curve becomes geometry dependent. In this intermediate region the water release curve is discontinuous due to the topologically different solutions obtained at different saturation. Even in simple cases such as those considered in §4 the simulations we have done show that the macroscopic features are strongly related to the geometry studied.

The resulting complexity and discontinuities in both the water release curve and the diffusivity requires some attention. The solutions obtained in §4 are based around a chosen initial condition. However, it is clear that, even for the two-dimensional case, the solutions pictured are not the only possible ones. A different initial condition would have resulted in a different solution structure. For example, the solution shown in figure 2 would be equally valid if the images were simply rotated by 90°. Similarly, the solutions pictured for the three-dimensional case, see figure 4, are equally valid if the solution is rotated by 90° about any of the three coordinate axis. Hence, in general, we expect that the calculated solution will be a combination of topologically different states and that the observed properties of the two fluids will be an average of all possible states.

The water release and diffusivity curves obtained for the sample geometries exhibit hysteresis. This hysteresis is entirely due to the non-linear behaviour of the fluid–fluid interface, i.e. for a given saturation there are multiple stationary solutions. For increasing saturation a different solution is obtained to decreasing saturation. In principle, other sources of hysteresis such as contact angle hysteresis could be included in the model. However, excluding these does not prevent the model from capturing the main observable effects of the two fluid–fluid interaction in a porous geometry.

Finally, we note that the ability to directly predict the water release curves directly from a porous geometry, for example soil, enables a much more precise set of macroscopic equations to be derived without the need for time consuming measurements. While we have demonstrated this method in using parameters appropriate to the flow of air and water in soil, it is applicable to a variety of two fluid systems. In the context of soil, this method, combined with image-based modelling, can be used as a tool to study the effects of different microscopic soil properties on the macroscopic behaviour. This in turn will directly feed back into how soil structure may be optimized in order to maximize flow and transport properties.

## Data accessibility

The corresponding author may be contacted for the Comsol implementation of the computational method described in this work.
